# Alkaline mineral complex supplementation alters gut microbiota and metabolic profiles and supports colonic health in finishing cattle

**DOI:** 10.3389/fmicb.2026.1822268

**Published:** 2026-06-02

**Authors:** Jia Li, Xingyu Liu, Li Gu, Xiaowan Liu, Yixin Wang, Hongrui Guo, Liping Gou, Jiancheng Qi, Zhicai Zuo

**Affiliations:** Key Laboratory of Animal Disease and Human Health of Sichuan Province, College of Veterinary Medicine, Sichuan Agricultural University, Chengdu, Sichuan, China

**Keywords:** alkaline mineral complex (AMC), colonic health, finishing cattle, gut microbiota, multi-omics analysis

## Abstract

**Introduction:**

The ruminant colon plays a critical role in intestinal homeostasis but is highly susceptible to dysfunction induced by high-concentrate diets during the finishing period. This study investigated the efficacy of alkaline mineral complex (AMC) against such colonic disturbances in finishing cattle.

**Methods:**

Fifty-four Simmental crossbred cattle were randomly allocated into the Ctrl group (*n* = 27) and AMC supplementation group (*n* = 27) over a 142-day feeding trial. Fecal consistency was recorded twice daily on 2 days per week in 10 randomly selected cattle per group, and on day 142, samples of blood, colonic tissue, mucosa, and contents were collected for serum biochemistry, histology, 16S rRNA gene sequencing, metabolomics, and proteomics.

**Results:**

AMC supplementation significantly improved fecal consistency, markedly reducing watery feces (*P* = 0.0082) while increasing normal feces (*P* = 0.0051). Serum biochemistry showed that AMC supplementation was associated with higher serum concentrations of Na^+^ (*P* = 0.0058), K^+^ (*P* = 0.0141), and total Ca (*P* = 0.0076), along with reduced diamine oxidase (DAO) activity (*P* < 0.0001) compared to the Ctrl group (*n* = 10 per group). Histological analysis showed that AMC supplementation reduced inflammation and increased goblet cells (*P* = 0.0013) (*n* = 3 per group). 16S rRNA gene sequencing demonstrated that AMC supplementation increased gut microbial α-diversity and reduced abundance of potentially pathogenic bacteria (*n* = 5 per group). Metabolomic analysis showed that AMC supplementation was associated with lower levels of Sph(d18:0) and ornithine, and with distinct alterations in sphingolipid metabolism and branched-chain amino acid (BCAA) degradation pathways (*n* = 5 per group). Proteomic profiling highlighted the enrichment of aldosterone-regulated sodium reabsorption and tight junction pathways (Ctrl, *n* = 4; AMC, *n* = 5). Using ELISA and spectrophotometric assays, we found that AMC supplementation significantly downregulated the levels of TNF-α (*P* = 0.0401) and IL-6 (*P* = 0.0104), while elevating GSH-Px activity (*P* = 0.0097) (*n* = 10 per group). The qPCR results indicated that AMC supplementation upregulated the mRNA expression of key intestinal barrier components *MUC2* (*P* = 0.0186), *ZO-1* (*P* = 0.0382), and transporters *AQP3* (*P* = 0.0156), *NBCe1* (*P* < 0.0001), and *ATP1A1* (*P* < 0.0001) (*n* = 3 per group).

**Discussion:**

Collectively, AMC supplementation improved fecal consistency and markers of colonic health. These benefits were accompanied by coordinated shifts in the gut microbiota, metabolic, and proteome, reduced inflammation, and enhanced expression of barrier and transport genes, suggesting a potential interplay between the microbiota, metabolome, and host mucosa.

## 1 Introduction

The ruminant colon plays a critical role in maintaining intestinal homeostasis (Jin et al., 2024), primarily by harboring a dense commensal microbiota, serving as a barrier via its epithelial layer, and absorbing water and electrolytes (Li et al., 2024; Negussie et al., 2022). In intensive beef production systems, the hindgut health of finishing cattle is frequently compromised by high-concentrate diets designed for rapid weight gain (Hou et al., 2024). Unlike the rumen, which possesses robust buffering capacity, the colon is vulnerable due to its limited buffering mechanisms and single-layered columnar epithelium (Gressley et al., 2011; Li et al., 2012; Hou et al., 2024). When undigested starch bypasses the rumen and undergoes excessive fermentation in the hindgut, the accumulation of volatile fatty acids (VFAs) lowers luminal pH, while the concomitant overgrowth of Gram-negative bacteria increases the release of lipopolysaccharides (LPS), together precipitating mucosal damage (Mao et al., 2012). This acidic, inflammatory microenvironment disrupts electrolyte transport and downregulates tight junction proteins (Borenshtein et al., 2008; Johansson and Hansson, 2013; Carter et al., 2021), ultimately leading to barrier failure and clinical diarrhea (Liu et al., 2014; Wang et al., 2017; Grimm et al., 2020). Consequently, maintaining colonic homeostasis is pivotal for sustaining systemic health and productivity in finishing cattle.

Nutritional interventions, particularly various feed additives, are commonly employed to mitigate gastrointestinal disturbances in ruminants. However, the benefits of single-component interventions are inconsistent and often accompanied by specific limitations (Golder et al., 2014). For instance, while specific trace minerals supplementation alleviates colonic oxidative stress and apoptosis (Samo et al., 2020), it might affect the absorption of other essential trace minerals (McMillen et al., 2022). Similarly, chemical buffers neutralize luminal acidity but often fail to improve growth performance (Tripathi et al., 2004; Colombo et al., 2022). Even microbial modulators, such as specific yeast strains, have been associated with elevated blood D-lactate levels despite their beneficial effects on microbiota composition (Carter et al., 2025). These limitations underscore the need for composite nutritional strategies capable of concurrently modulating the microbiome, reinforcing the physical barrier, and restoring physiological homeostasis.

As a potential solution, the alkaline mineral complex contains probiotics like *Bacillus subtilis* and *Bacillus licheniformis*, along with essential minerals such as sodium, potassium, zinc, germanium and metasilicic acid (Qi et al., 2024). The probiotic constituents may directly modulate the colonic microbiota and mucosal immune responses (Aykut et al., 2024), while the mineral components could contribute to luminal buffering capacity, serve as cofactors for epithelial repair processes, and influence systemic electrolyte balance (Polli et al., 2025), thereby targeting the multiple facets of colonic dysfunction induced by high-concentrate diets. Previous studies have demonstrated AMC’s capacity to scavenge reactive oxygen species (ROS) in humans (Shin et al., 2018; Zhu et al., 2021), alleviate stress-induced intestinal inflammation in weaned piglets (Chen et al., 2022; Chen J. et al., 2023), and improve respiratory health and growth performance in transported calves (Qi et al., 2024). However, these prior studies have focused primarily on stress-induced conditions such as weaning in piglets or transport in calves, and have examined outcomes related to growth performance, respiratory health, or intestinal inflammation. Whether AMC can protect the colonic environment of finishing cattle against the insult of high-concentrate feeding, a condition fundamentally different from stress-induced models, remains unexplored. Furthermore, no study has yet employed an integrated multi-omics approach to comprehensively characterize the colonic response to AMC in finishing cattle. We hypothesized that AMC supplementation would preserve colonic health by ameliorating the inflammatory microenvironment, remodeling the dysbiotic microbiota, and activating epithelial repair pathways. Therefore, this study employed an integrated multi-omics approach, combined with histological, serum biochemical, and gene expression analyses, to investigate how AMC supplementation modulates the colonic microbiome, metabolome, and proteome, and to explore their potential associations with inflammation, barrier integrity, ion transport, and fecal consistency in finishing cattle.

## 2 Materials and methods

### 2.1 Animal ethics statement

All animal experiments were approved by the Institutional Animal Care and Use Committee of Sichuan Agricultural University (Approval Code: 20240501).

### 2.2 Animal experiment

The animal experiment was conducted at Caijiashan Breeding Company, Qu County, Dazhou, Sichuan Province. A total of 54 healthy, crossbred Simmental bulls, approximately 14 months of age, were randomly assigned to two groups (*n* = 27 per group): the control (Ctrl) group and AMC supplementation group (AMC, Cat No: Q/NEL002-2019, Beijing Jnnail Biological Technology Co., Ltd., Beijing, China). Initial body weight (mean ± SD) was 371.83 ± 20.76 kg in the Ctrl group and 388.28 ± 36.89 kg in the AMC group, and did not differ between groups (*P* > 0.05). The individual animal was treated as the experimental unit. Cattle were housed in 6 pens (3 pens per group, 9 cattle per pen) with *ad libitum* access to water. The total experimental period was 142 days.

All cattle were fed a total mixed ration (TMR) with a concentrate-to-roughage ratio of 60:40, delivered twice daily at 09:30 and 16:30. The TMR formulation met or exceeded the nutritional requirements for beef cattle (Nutrient requirements of beef cattle, 8th revised edition, National Academies Press, 2015). For the AMC group, the daily AMC dose (20 g per head) was first mixed into a portion of the TMR and offered to allow priority consumption; once this was fully consumed, the remaining TMR was provided. No refusal of the AMC-containing feed portion was observed throughout the experimental period. Each head in the AMC group received a target dose of 20 g of AMC per day, while the Ctrl group was fed only the base TMR. The composition and quality specifications of AMC are provided in Supplementary Table 1. The detailed TMR composition and nutritional values are provided in Supplementary Table 2.

### 2.3 Sample collection

On day 142, before morning feeding, 30 mL of blood was collected from the jugular vein of 10 randomly selected cattle from each group into vacuum blood collection tubes. The samples were allowed to stand at room temperature for 30 min, then centrifuged at 3,000 × g for 8 min at 4°C to separate the serum. The serum was aliquoted into 1.5 mL tubes and stored at -80°C for subsequent biochemical analysis.

Following blood collection, the same 10 cattle from each group were slaughtered. After slaughter, distal colonic contents were immediately collected under sterile conditions and rapidly frozen at -80°C for subsequent 16S rRNA gene sequencing and metabolomic analysis. A 20-cm segment of distal colon tissue was excised, thoroughly flushed with cold sterile saline three times to remove residual intestinal contents. The tissue was divided into two portions: one was fixed in 4% paraformaldehyde for histological evaluation, while the other was scraped for mucosal tissue, rapidly frozen in liquid nitrogen, and stored at -80°C for gene and protein level analysis. Histomorphometric measurements (mucosal thickness and goblet cell counts) were performed in three randomly selected animals per group.

### 2.4 Fecal consistency and serum biochemical analysis

Throughout the experimental period, fecal consistency was evaluated twice daily (morning and afternoon) on 2 days per week. At each evaluation, 10 cattle per group were randomly selected for scoring, using the system (1–5) proposed by Zaaijer and Noordhuizen (2003). Scores were redefined into three categories: score 1–2 for hard and firm feces, score 3 for normal feces, and score 4–5 for watery feces. Because cattle were group-housed, individual identification was not feasible at each scoring event; therefore, the animals evaluated varied across time points. All assessments were performed by the same trained observer to ensure scoring consistency.

Serum electrolytes were quantified using a BS-2000M automatic biochemical analyzer (Mindray Biomedical Electronics Co., Ltd., Shenzhen, China), according to the manufacturer’s protocols.

Serum diamine oxidase (DAO) and D-lactic acid (D-LA) levels were measured using enzyme-linked immunosorbent assay (ELISA) kits (DAO, Cat No: MM-5089102; D-LA, Cat No: MM-5132902; Jiangsu Meimian Industrial Co., Ltd., Jiangsu, China). All assays followed the manufacturer’s protocols, and absorbance was read using a microplate reader. Concentrations were calculated by interpolation from standard curves.

### 2.5 Histopathological examination and AB-PAS staining

Colonic tissue samples fixed in 4% paraformaldehyde were processed for histological analysis. The tissues were dehydrated through a graded ethanol series, cleared in xylene, infiltrated with paraffin, and embedded in paraffin blocks. Continuous 5 μm sections were prepared using a Leica RM2235 microtome (Leica Microsystems, Germany) and mounted onto glass slides. Sections were stained with hematoxylin-eosin (H&E) for histological observation. Colonic mucosal thickness and morphology were measured using a Leica DM500 optical microscope (Leica Microsystems, Germany) with a calibrated eyepiece micrometer.

For goblet cell quantification, additional sections were stained with Alcian blue periodic acid-Schiff (AB-PAS). Sections were deparaffinized, rehydrated, stained with Alcian blue, treated with 1% periodate oxidation, Schiff’s reagent, and hematoxylin counterstaining, followed by dehydration, clearing, and mounting. Images were captured using a Leica DM1000 microscope (Leica Microsystems, Germany) equipped with a digital imaging system. The number of goblet cells in each crypt was counted blindly.

### 2.6 Inflammatory cytokine and oxidative stress analysis

The colonic mucosa samples were homogenized in pre-chilled phosphate-buffered saline (PBS, pH 7.4) into a uniform slurry using an automatic grinder (MB-LD48S, Beijing Boaojuhe Technology Co., Ltd., Beijing, China), and centrifuged at 12,000 × g for 15 min at 4°C. The supernatants were collected and aliquoted for subsequent analysis.

Inflammatory Cytokines: Tumor necrosis factor-alpha (TNF-α, Cat No: MM-156801), interleukin-6 (IL-6, Cat No: MM-3473601), and interleukin-1β (IL-1β, Cat No: MM-3694901) were quantified using bovine-specific ELISA kits (Jiangsu Meimian Industrial Co., Ltd., Jiangsu, China) in triplicate.

Oxidative Stress Markers: Total antioxidant capacity (T-AOC, Cat No: A015-2-1), superoxide dismutase (SOD) activity (Cat No: A001-3-2), catalase (CAT) activity (Cat No: A007-1-1), glutathione peroxidase (GSH-Px) activity (Cat No: A005-1-2), and malondialdehyde (MDA) content (Cat No: A003-1-2) were measured using commercial assay kits (Nanjing Jiancheng Bioengineering Institute, Nanjing, China), following the manufacturer’s protocols. Measurements were performed using a spectrophotometric method with a microplate reader (BioTek Instruments, United States). The activity of antioxidants (SOD, CAT, GSH-Px) was expressed as U/mg protein, while T-AOC (mmol/g protein) and MDA levels (nmol/mg protein) were normalized to total protein concentration, determined using the BCA protein assay kit (Nanjing Jiancheng Bioengineering Institute, Nanjing, China).

### 2.7 Gene expression analysis

Total RNA was extracted from bovine colonic mucosal tissues using TRIzol reagent (TransGen Biotech, Beijing, China), following the manufacturer’s protocol. Tissue samples were homogenized in TRIzol, followed by phase separation with chloroform, RNA precipitation with isopropanol, and ethanol washing. The RNA pellets were air-dried and dissolved in RNase-free water. RNA concentration and purity were measured using a spectrophotometer. First-strand complementary DNA (cDNA) was synthesized using the TransScript Uni All-in-One First-Strand cDNA Synthesis SuperMix (Cat No: AU341-02; TransGen Biotech, Beijing, China) with 1 μg of total RNA as the template, according to the manufacturer’s instructions. Quantitative PCR was performed using PerfectStart Green qPCR SuperMix (Cat No: AQ601-01-V2; TransGen Biotech, Beijing, China) on a QX300 real-time PCR system (Sichuan Jialamei Technology Co., Ltd., China). The amplification program included an initial denaturation at 94°C for 30 s, followed by 45 cycles of denaturation at 94°C for 5 s and annealing/extension at 60°C for 30 s. Reactions were run in triplicate. β-actin was used as the internal control for normalization. The expression stability of β-actin was confirmed by the absence of significant differences in Ct values between the Ctrl and AMC groups across all target genes analyzed (*P* > 0.05). Gene expression levels were calculated using the 2^–Δ^ ^Δ^ *^CT^* method. All primer sequences used in this study are provided in Supplementary Table 3.

### 2.8 16 S rRNA gene sequencing and analysis

Colonic content samples stored at -80°C were transported on dry ice within 3 days to Novogene Biotechnology Co., Ltd. (Beijing, China) for 16S rRNA gene sequencing and analysis. Genomic DNA was extracted from colonic contents using the TIANamp Stool DNA Kit (TIANGEN Biotech, Beijing, China) and assessed by a NanoDrop 2000 spectrophotometer (Thermo Fisher Scientific, United States). The V3–V4 hypervariable region of the 16S rRNA gene was amplified using primers 341F (5’-CCTAYGGGRBGCASCAG-3’) and 806R (5’-GGACTACNNGGGTATCTAAT-3’) with Phusion High-Fidelity PCR Master Mix (New England Biolabs, United States). The PCR protocol included an initial denaturation at 98°C for 1 min, followed by 30 cycles (98°C/10 s, 50°C/30 s, 72°C/30 s), and a final extension at 72°C for 5 min. PCR products were inspected using 2% agarose gel electrophoresis. Qualified products were purified using magnetic beads, quantified enzymatically, and mixed in equiproportional amounts based on their concentration. The mixture was checked again via 2% agarose gel electrophoresis, and the target bands were recovered. Libraries were constructed and subsequently quantified using Qubit and Q-PCR. After qualification, the libraries were sequenced on an Illumina NovaSeq 6,000 platform (250 bp paired-end reads). For data processing, paired-end reads were merged using FLASH (v1.2.11) after barcode and primer truncation. The merged tags underwent quality filtering via fastp (v0.23.1) and chimera removal against the SILVA database (v138.1) to obtain effective tags. These tags were subsequently denoised using the DADA2 module in QIIME2 (v2022.2) to generate Amplicon Sequence Variants (ASVs). Taxonomy was assigned to ASVs using the SILVA 138.1 database, and diversity analyses were performed using QIIME2 and R software (v4.2.1).

### 2.9 Metabolomics analysis

Non-targeted metabolomics was conducted by Shanghai Biotree Biotech Co., Ltd. (Shanghai, China). Approximately 25 mg of colonic content was weighed into an EP tube, followed by the addition of two homogenization beads and 500 μL of pre-chilled extraction solution (methanol: acetonitrile: water = 2:2:1, v/v) containing isotope-labeled internal standards. After vortexing for 30 s, samples were homogenized (35 Hz, 4 min) and sonicated (5 min, ice-water bath). This homogenization-sonication cycle was repeated three times. Extracts were incubated at -40°C for 1 h and centrifuged at 12,000 rpm for 15 min at 4°C. Supernatants were transferred to LC-MS vials for analysis, and pooled quality control (QC) samples were prepared by mixing aliquots of all supernatants. Metabolomic profiling was performed on a Vanquish UHPLC system (Thermo Fisher Scientific, United States) equipped with a Waters ACQUITY UPLC BEH Amide column (2.1 mm × 50 mm, 1.7 μm), coupled to an Orbitrap Exploris 120 mass spectrometer (Thermo Fisher Scientific, United States) operated under Xcalibur (v4.4). Raw MS data were converted to mzXML format using ProteoWizard, and peak detection, extraction, alignment, and integration were performed using an XCMS-based R pipeline. Metabolites were identified by matching against the BiotreeDB (V3.0) database using in-house R scripts. Prior to statistical analysis, the data were log10-transformed and Pareto-scaled. Missing values were imputed using the minimum value method. All samples were analyzed in a single batch.

### 2.10 Proteomics analysis

Proteomic analysis was conducted by Shanghai Biotree Biotech Co., Ltd. (Shanghai, China). One Ctrl sample failed quality control due to insufficient peptide identifications and was excluded, resulting in four Ctrl samples and five AMC samples for subsequent analysis. Colonic mucosa samples were homogenized in RIPA lysis buffer with stainless steel beads and sonicated in an ice-water bath. Following centrifugation, supernatants were collected and protein concentrations determined using a BCA Protein Assay Kit. For each sample, 30 μg of protein underwent denaturation, reduction, alkylation, enzymatic digestion, and desalting according to standard protocols. Peptides were concentrated by vacuum centrifugation, reconstituted in mobile phase A, and 200 ng of peptides were separated on a C18 reversed-phase column using a nanoElute2 UPLC system coupled to a timsTOF Pro2 instrument (Bruker, Germany) equipped with a nanoelectrospray ion source. A 45-min gradient was applied, and data-independent acquisition (DIA) was performed in PASEF mode over an m/z range of 350–1,250. DIA data were processed using a label-free quantification workflow in Spectronaut software with the Pulsar search engine against the latest release of the UniProt *Bos taurus* protein database. Carbamidomethylation of cysteine was set as a fixed modification, while methionine oxidation and protein N-terminal acetylation were defined as variable modifications. Trypsin was specified as the proteolytic enzyme with up to two missed cleavages allowed. Mass tolerances for precursors and fragments were set at 20 ppm, and false discovery rates (FDRs) for peptide-spectrum matches (PSMs) and peptides were controlled at 1%. All samples were processed and analyzed in a single batch. Prior to statistical analysis, protein intensities were log2-transformed and median-centered. Proteins with > 50% missing values within each group were excluded.

### 2.11 Statistical analysis

All data are presented as mean ± standard error of the mean (SEM), and statistical analyses were performed using SPSS 26.0 (IBM, NYC, United States). Graphs were generated with GraphPad Prism 9.3 (GraphPad Software, CA, United States), Novomagic,^1^ or Lims2,^2^ unless otherwise specified. Normality of data distribution was assessed using the Shapiro-Wilk test, and homogeneity of variances was evaluated using Levene’s test. Data meeting both assumptions were analyzed using Student’s *t*-test; otherwise, the Mann-Whitney U test was applied. *P* < 0.05 was considered statistically significant. Fecal consistency data were analyzed using a generalized linear mixed model (GLMM). In this model, each fecal category served as the dependent variable, with “Group” (Ctrl or AMC) as a fixed factor. “Time points” was treated as a repeated-measures factor with a first-order autoregressive [AR(1)] covariance structure. Factors with *P* < 0.05 were considered to have significant effects on the outcomes. For microbiota, α diversity indices (chao1 and observed_features) were compared using Student’s unpaired *t*-test, β diversity was assessed by principal coordinates analysis (PCoA) and non-metric multidimensional scaling (NMDS) based on Bray-Curtis distances, and statistical significance of community composition differences between groups was evaluated using PERMANOVA (*R*^2^ and *P-*values reported). Differentially abundant taxa were identified using linear discriminant analysis effect size (LEfSe, LDA score > 2, *P* < 0.05), and microbial functional prediction was conducted with Tax4Fun. Metabolomic data were analyzed by orthogonal partial least squares-discriminant analysis (OPLS-DA) with model validation by 200 permutation tests; differential metabolites (DEMs) were defined as those with variable importance in projection (VIP) > 1 and *P* < 0.05, and pathway enrichment was assessed using the Kyoto Encyclopedia of Genes and Genomes (KEGG) database. Proteomic analysis identified differentially expressed proteins (DEPs) with foldchange ≥ 1.2 or ≤ 0.83 and *P* < 0.05 (Student’s *t*-test or chi-test), a threshold selected to capture a broader range of biologically meaningful candidates in this exploratory multi-omics study. Gene Ontology (GO) annotation with UniProt, functional classification via KEGG, and Protein-protein interaction (PPI) networks were constructed using the STRING database (v12.0) with a minimum interaction score of 0.4 (medium confidence), and visualized using Cytoscape (v3.9.1). KEGG pathways were generated using Pathview. Correlation analyses were conducted using Spearman’s method to assess associations among microbial taxa, metabolites, and host indicators; significant correlations (| R| > 0.5, *P* < 0.05) were visualized with the corrplot and pheatmap R packages.

## 3 Results

### 3.1 AMC supplementation improves fecal consistency and colonic health

**FIGURE 1:**
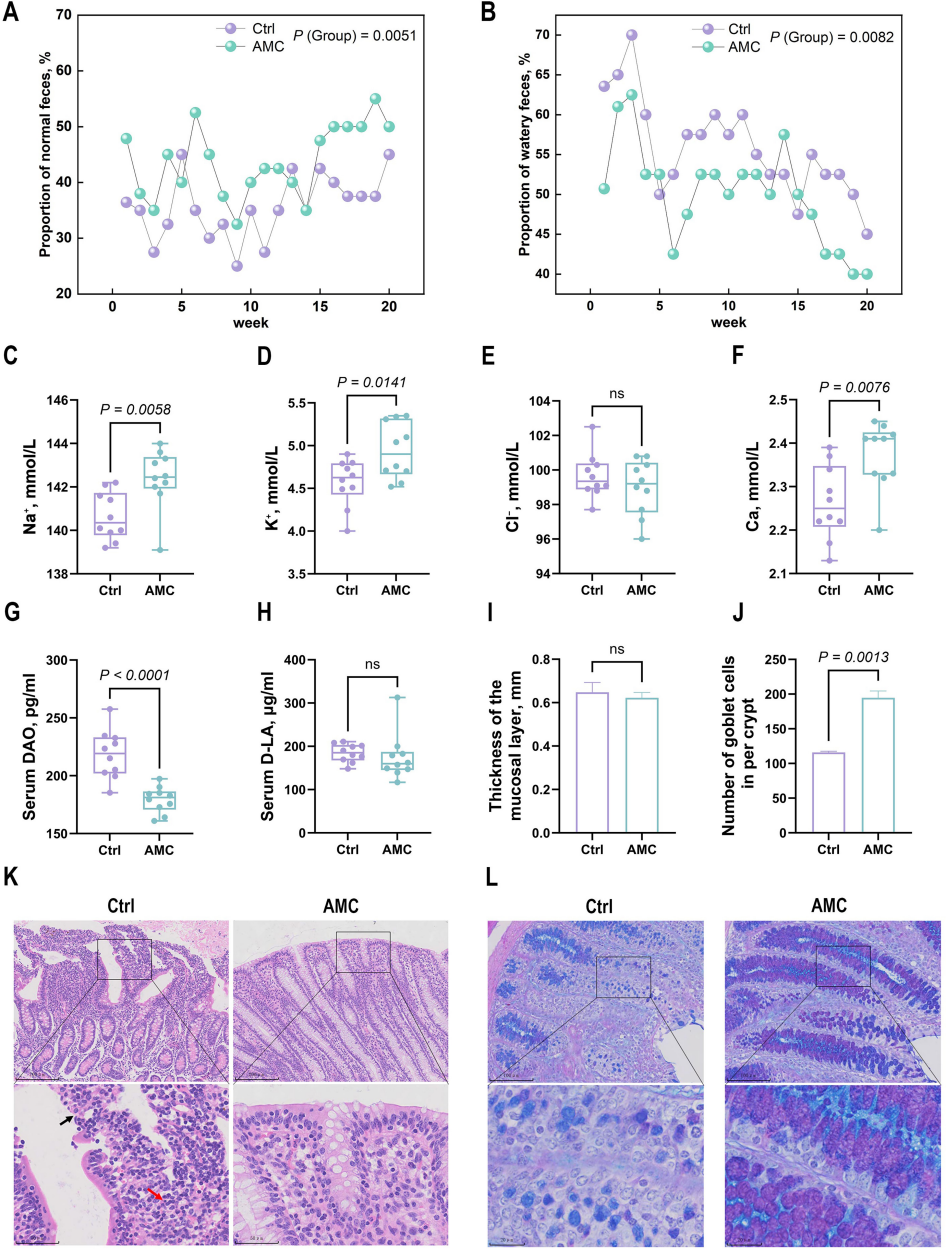
AMC supplementation improves fecal score, serum electrolytes, and colonic morphology. **(A)** Weekly frequency of normal feces (*n* = 10). **(B)** Weekly frequency of watery feces (*n* = 10). **(C–F)** Serum electrolytes: Na^+^ **(C)**, K^+^ **(D)**, Cl^+^ **(E)**, Ca **(F)** (*n* = 10). **(G)** Serum diamine oxidase (DAO) activity (*n* = 10). **(H)** Serum D-lactic acid (D-LA) concentration (*n* = 10). **(I)** Thickness of the colonic mucosal layer (*n* = 3). **(J)** Number of goblet cells per crypt (*n* = 3). **(K)** Representative images of H&E staining of colonic tissue. Scale bars: 20, 50 μm. Red arrows indicate inflammatory cell infiltration in the mucosal surface; black arrows indicate necrosis and exfoliation of surface epithelial cells. **(L)** Representative images of AB-PAS staining of colonic tissue Scale bars: 100, 20 μm.

AMC supplementation significantly improved fecal consistency. The AMC group demonstrated a markedly higher frequency of normal feces (*P* = 0.0051) and a lower frequency of watery feces (*P* = 0.0082) relative to the Ctrl group (Figures 1A,B). Consistent with the improved fecal consistency, the AMC group had higher serum concentrations of Na^+^ (*P* = 0.0058), K^+^ (*P* = 0.0141), and total Ca (*P* = 0.0076) (Figures 1C–F). Concurrently, intestinal barrier function was fortified, as evidenced by a substantial reduction in serum DAO activity (*P* < 0.0001) despite a non-significant change in D-LA levels (*P* > 0.05) (Figures 1G,H). Histological analyses further substantiated these functional benefits. Although AMC supplementation did not affect mucosal thickness (*P* > 0.05), it did preserve colonic architecture by maintaining intact epithelial layers and limiting inflammatory infiltration, in stark contrast to the extensive epithelial necrosis, exfoliation, and immune cell influx observed in the Ctrl group (Figures 1I,K). Moreover, AMC supplementation significantly augmented goblet cell density per crypt (*P* = 0.0013) (Figures 1J,L).

### 3.2 AMC supplementation modulates colon microbiota composition and function

**FIGURE 2:**
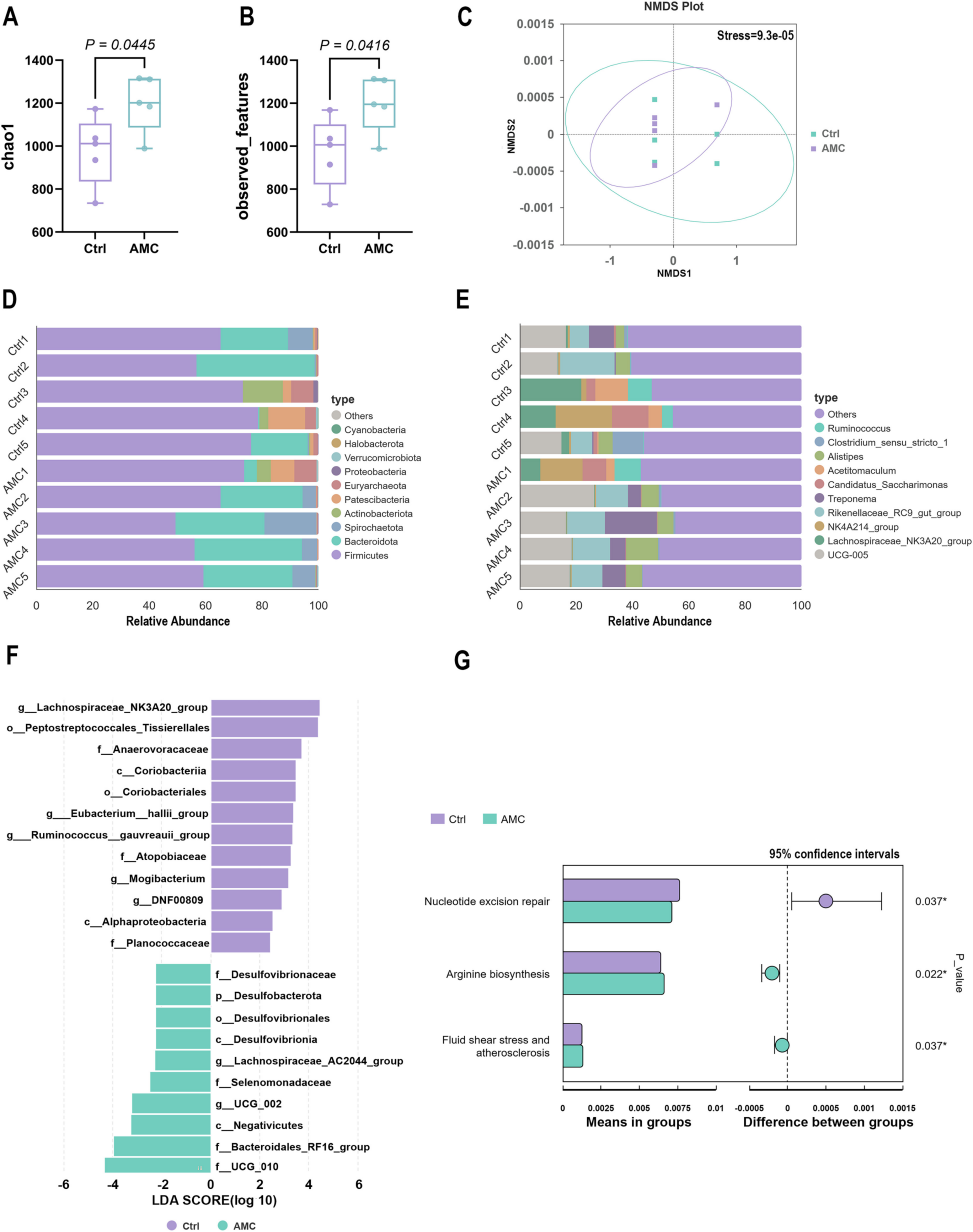
AMC supplementation alters the colonic microbiota (*n* = 5). **(A,B)** α-Diversity indices: chao1 **(A)**, observed_features **(B)**. **(C)** Non-metric multi-dimensional scaling (NMDS) plot based on bray-Curtis distance (Stress = 9.3e-05). **(D)** Relative abundance of bacterial communities at the phylum level. **(E)** Relative abundance of bacterial communities at the genus level. **(F)** Linear discriminant analysis effect size (LEfSe) histogram. **(G)** Relative abundance of predicted bacterial KEGG pathways (Level 3).

After quality control, a total of 826,470 clean reads were obtained, averaging 82,647 ± 3,077 reads per sample (Supplementary Table 4). The results revealed that AMC supplementation significantly enhanced the richness of the colonic microbiota, as indicated by increased chao1 (*P* = 0.0445) and observed_features (*P* = 0.0416) indices (Figures 2A,B). PCoA (Supplementary Figure 1) and NMDS (Stress = 9.3e-05; Figure 2C) showed no clear separation between groups, and PERMANOVA confirmed that the overall microbial community composition did not differ significantly (*R*^2^ = 0.112, *P* = 0.21). Taxonomic analysis identified *Firmicutes* and *Bacteroidota* as the dominant phyla in both the Ctrl and AMC groups (Figure 2D), with *UCG-005*, *Rikenellaceae RC9 gut* group, and *NK4A214* group representing highly abundant genera common to both groups (Figure 2E). LEfSe analysis was performed across all taxonomic ranks from phylum to genus. In the Ctrl group, enriched taxa included the genera *Lachnospiraceae* NK3A20 group, *Eubacterium hallii* group, *Mogibacterium*, the families *Anaerovoracaceae* and *Atopobiaceae*, the orders *Peptostreptococcales Tissierellales* and *Coriobacteriales*, and the classes *Alphaproteobacteria* and *Coriobacteriia*. In the AMC group, enriched taxa included the genera *UCG-010* and *UCG-002*, the family *Bacteroidales* RF16 group, and the class *Negativicutes* (LDA > 2, *P* < 0.05) (Figure 2F). Functional prediction using Tax4Fun revealed distinct metabolic profiles between the two groups. Compared with the Ctrl group, the AMC group showed significant enrichment of the arginine biosynthesis and fluid shear stress and atherosclerosis pathways, whereas the nucleotide excision repair pathway was significantly enriched in the Ctrl group (Figure 2G).

### 3.3 AMC Supplementation alters colon metabolite profile and key pathways

**FIGURE 3:**
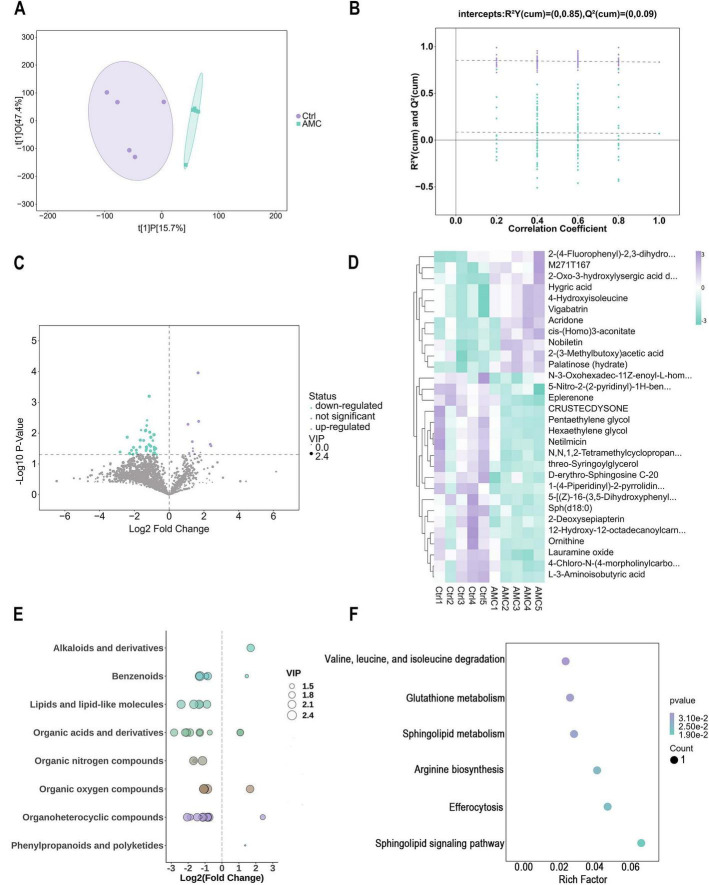
AMC supplementation alters the metabolomic profile (*n* = 5). **(A)** Scores plot of orthogonal partial least square discriminant analysis (OPLS-DA). **(B)** Permutation test plot (Intercepts: *R*^2^Y = 0.85, Q^2^ = 0.09). **(C)** Volcano plot of differential metabolites. **(D)** Heatmap of differential metabolites (top 30). **(E)** Bubble plot of differential metabolites classification. **(F)** Pathway enrichment bubble plot.

Non-targeted metabolomic analysis identified a total of 2,649 metabolites in the colonic content. OPLS-DA demonstrated clear separation between the Ctrl and AMC groups, with distinct intra-group clustering (Figure 3A). The model was rigorously validated through 200 permutation tests (intercepts: *R*^2^Y = 0.85, Q^2^ = 0.09), confirming its robustness, accuracy, and absence of overfitting (Figure 3B). Based on the criteria of VIP > 1 and *P* < 0.05, a volcano plot revealed significant alterations in metabolite levels, with 34 metabolites downregulated and 11 metabolites upregulated in the AMC group compared to the Ctrl group (Figure 3C). A heatmap visualized the clustering of the top 30 DEMs and illustrated their abundance patterns in response to AMC supplementation (Figure 3D). These DEMs were classified into 8 categories and primarily belonging to lipids, organic acids, and benzenoids (Figure 3E). KEGG pathway enrichment analysis of these DEMs identified 6 pathways affected by AMC supplementation: sphingolipid metabolism, branched-chain amino acid (BCAA: valine, leucine, and isoleucine) degradation, arginine biosynthesis, glutathione metabolism, sphingolipid signaling pathway, and efferocytosis (Figure 3F). The differential abundance score (DAS) indicated an overall lower abundance of metabolites within the significantly enriched pathways in the AMC group (Supplementary Figure 2).

### 3.4 AMC supplementation alters protein expression to exert protective effects

**FIGURE 4:**
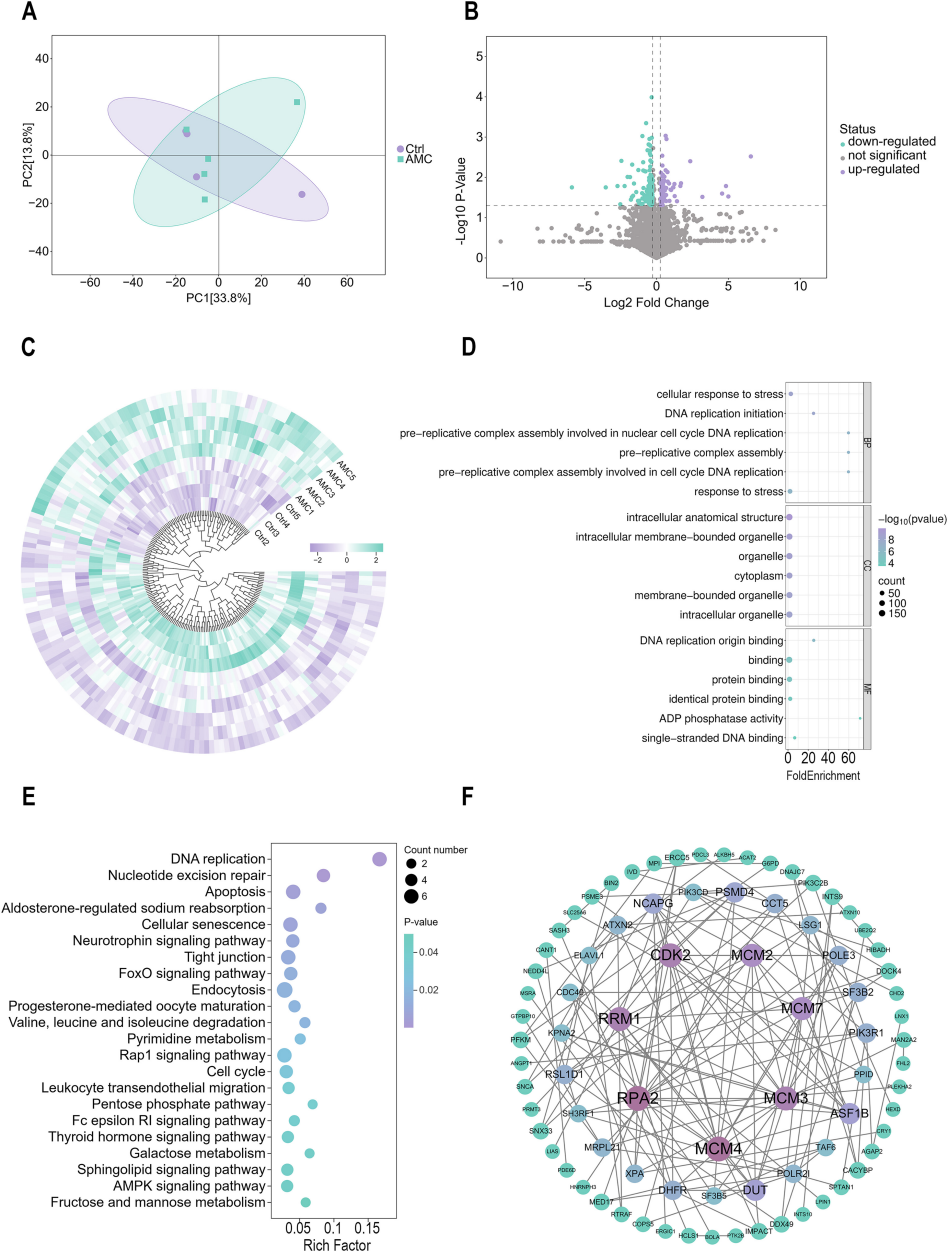
AMC supplementation alters the proteomic profile (Ctrl, *n* = 4; AMC, *n* = 5). **(A)** Principal component analysis (PCA) of protein expression profiles. **(B)** Volcano plot of differentially expressed proteins. **(C)** Hierarchical clustering heatmap of differential proteins (DEPs). **(D)** Gene Ontology (GO) enrichment analysis of differential proteins. **(E)** KEGG pathway enrichment analysis of DEPs. **(F)** Protein-protein interaction (PPI) network of DEPs.

To better understand the molecular mechanisms underlying AMC’s protective effects, we performed a proteomic analysis to investigate protein expression changes in the colonic mucosa of the Ctrl and AMC groups. A total of 8,369 proteins were identified. Principal component analysis (PCA) did not show significant separation between the two groups (Figure 4A). Based on a selection criterion of foldchange ≥ 1.2 or ≤ 0.83 and *P* < 0.05 (or chi-test *P* < 0.05), a volcano plot identified proteins with significant differential expression (Figure 4B). In comparison to the Ctrl group, 119 proteins were significantly downregulated, and 77 proteins were significantly upregulated in the AMC group. A heatmap was generated to visualize the DEPs (Figure 4C). GO analysis of the DEPs revealed significant enrichment in biological processes (BPs), cellular components (CCs), and molecular functions (MFs). The BPs mainly involved stress response, pre-replicative complex assembly in DNA replication, and cellular response to stress. The CCs were primarily associated with intracellular organelles, membrane-bound organelles, and the cytoplasm. The MFs included single-stranded DNA binding, ADP phosphatase activity, protein binding, and DNA replication origin binding (Figure 4D). KEGG pathway analysis further revealed significant enrichment in 22 pathways, including DNA replication, nucleotide excision repair, apoptosis, aldosterone-regulated sodium reabsorption, cellular senescence, neurotrophin signaling, and tight junction (Figure 4E). PPI network analysis identified a network of 81 proteins and 179 interaction relationships, with key hub proteins including MCM4, RPA2, CDK2, MCM3, MCM2, MCM7, and RRM1 (Figure 4F).

**FIGURE 5:**
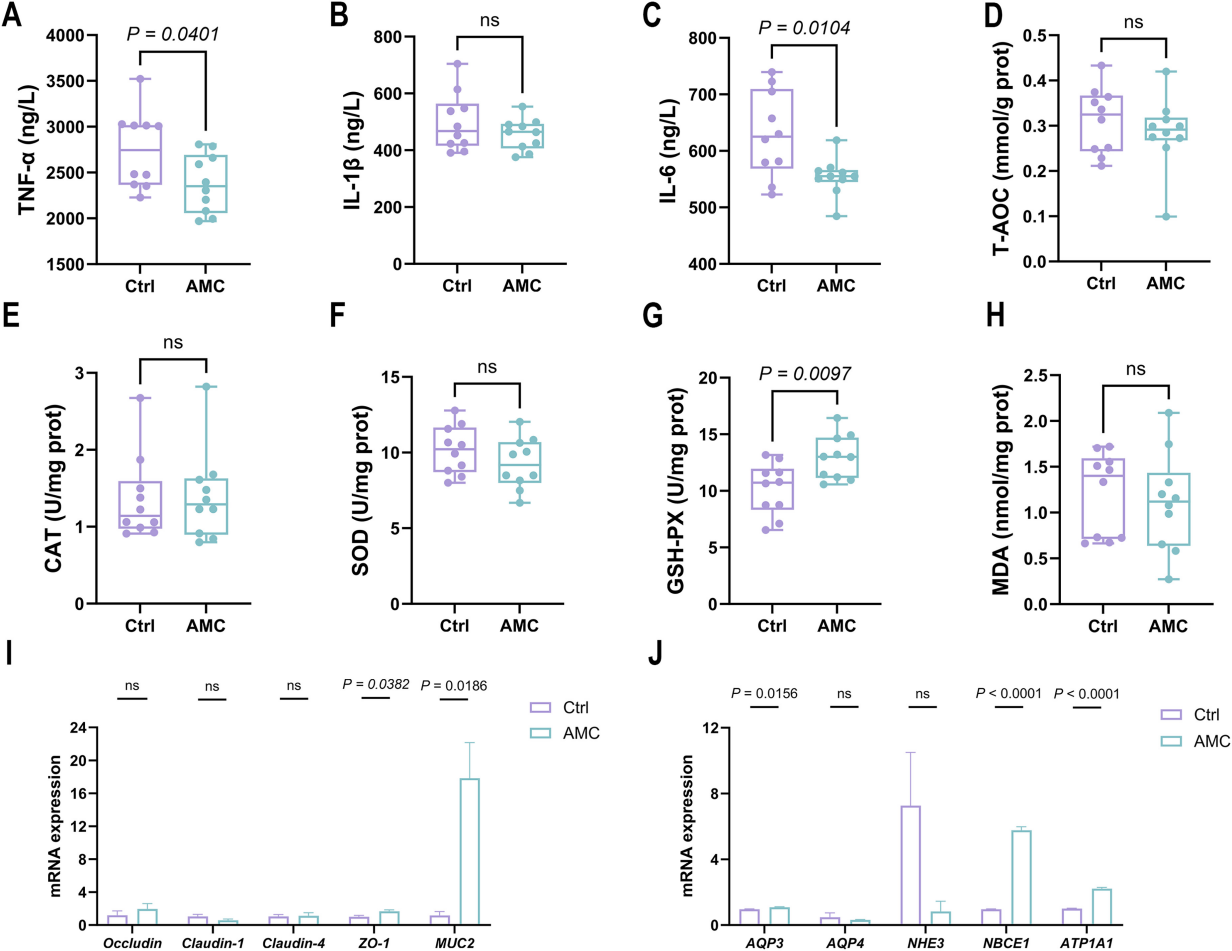
AMC supplementation improves anti-inflammatory and antioxidant capacity, barrier function, and transport activity. **(A–C)** Concentrations of pro-inflammatory cytokines in colonic mucosa: tumor necrosis factor-alpha (TNF-α) **(A)**, interleukin-1β (IL-1β) **(B)**, and interleukin-6 (IL-6) (*n* = 10). **(D–H)** Colonic mucosal antioxidant capacity and oxidative stress markers: total antioxidant capacity (T-AOC, **D**), catalase activity (CAT, **E**), superoxide dismutase activity (SOD, **F**), glutathione peroxidase activity (GSH-Px, **G**), and malondialdehyde content (MDA, **H**) (*n* = 10). **(I)** Relative mRNA expression of colonic tight junction proteins and mucin (MUC2, Occludin, Claudin-1, Claudin-4, ZO-1) (*n* = 3). **(J)** Relative mRNA expression of colonic ion and water transporters (AQP3, AQP4, NHE3, NBCe1, ATP1A1) (*n* = 3).

Complementing the proteomic results, molecular and physiological analyses provided functional validation. At the biochemical level, the AMC group exhibited significant mitigation of inflammatory and oxidative stress, marked by reduced levels of TNF-α (*P* = 0.0401) and IL-6 (*P* = 0.0104), alongside elevated GSH-Px activity (*P* = 0.0097). No significant changes were observed in the levels of IL-1β, T-AOC, CAT, SOD, and MDA (*P* > 0.05) (Figures 5A–H). qPCR results revealed that AMC supplementation significantly upregulated the expression of the tight junction protein *ZO-1* (*P* = 0.0382) and the mucin *MUC2* (*P* = 0.0186), whereas the mRNA levels of *Occludin*, *Claudin-1*, and *Claudin-4* remained unaltered (*P* > 0.05) (Figure 5I). Concurrently, AMC supplementation significantly enhanced the mRNA expression of ion and water transporters *AQP3* (*P* = 0.0156), *NBCe1* (*P* < 0.0001), and *ATP1A1* (*P* < 0.0001), with no significant effect detected on *AQP4* and *NHE3* (*P* > 0.05) (Figure 5J).

### 3.5 Integrating multi-omics to uncover mechanistic insights

**FIGURE 6:**
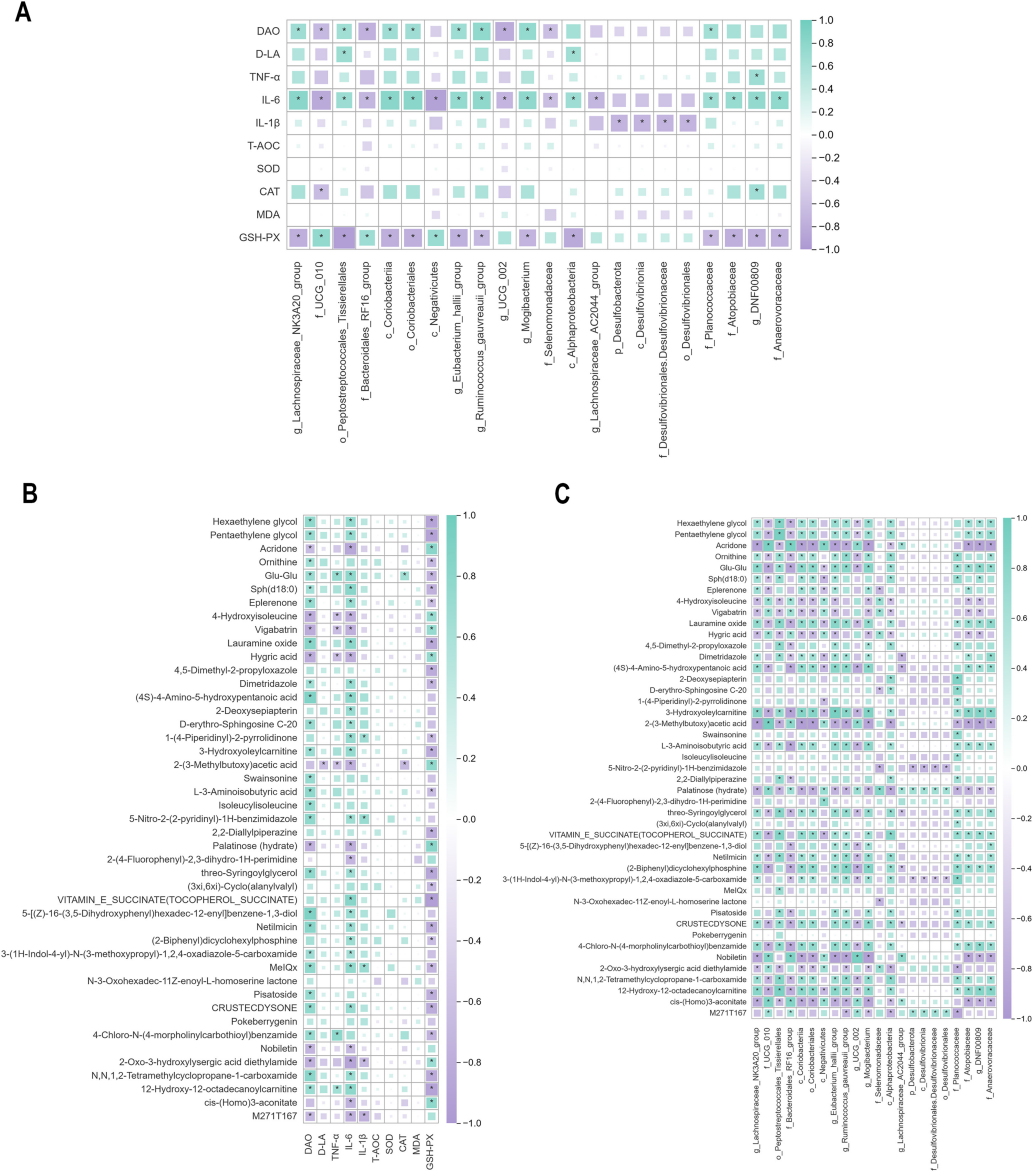
Correlations among microbiota, metabolites, and host health. **(A)** Spearman correlation heatmap between differential microbiota and host physiological indicators of barrier function, inflammation, and oxidative stress. **(B)** Spearman correlation heatmap between differential metabolites and host physiological indicators of inflammation, and oxidative stress. **(C)** Spearman correlation heatmap between differential metabolites and differential microbiota. Purple, positive correlations; Green, negative correlations, *P < 0.05.

To explore associations across omics layers, we performed Spearman correlation analyses integrating the microbiome, metabolome, and host physiological indicators. This analysis identified significant associations between specific microbial taxa and host health indicators: *Alphaproteobacteria* and *Mogibacterium* were positively correlated with IL-6 and negatively with GSH-Px (Figure 6A). Expanding this network, a correlation heatmap integrating microbial taxa with DEMs revealed that the metabolite Sph(d18:0) was positively correlated with serum DAO and IL-6, and negatively with GSH-Px. Similarly, ornithine and L-3-aminisobutyric acid correlated positively with DAO and negatively with GSH-Px activity (Figure 6B). Furthermore, these two metabolites were positively correlated with *Alphaproteobacteria* and *Mogibacterium*, while Sph(d18:0) showed a positive correlation with *Alphaproteobacteria* (Figure 6C).

**FIGURE 7:**
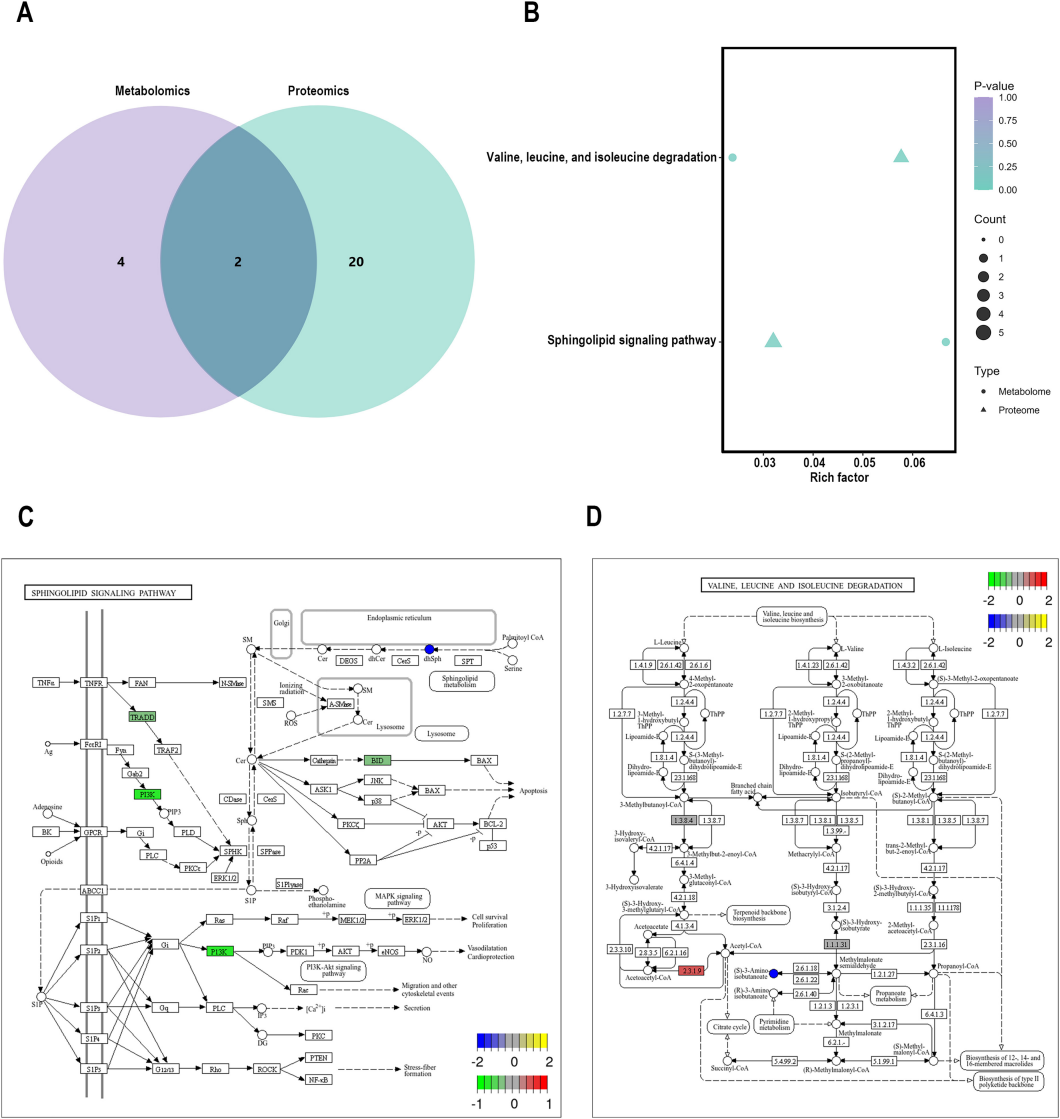
Integrated multi-omics reveals two core regulatory pathways. **(A)** Venn diagram of KEGG enrichment from metabolomic and proteomic analyses. **(B)** Shared KEGG pathways between proteomic and metabolomic analyses. **(C,D)** Visualization of DEPs and DEMs involved in the sphingolipid signaling pathway and BCAA degradation: The color gradient represents the log_2_ fold change ratio. Red indicates upregulated DEPs, green indicates downregulated DEPs, yellow indicates upregulated DEMs, and blue indicates downregulated DEMs in the AMC group.

Joint pathway analysis of the proteome and metabolome identified two convergent mechanisms: sphingolipid signaling pathway and BCAA degradation (Figures 7A,B). In the sphingolipid signaling pathway, DEPs (PIK3CD, PIK3R1, BID, TRADD) were downregulated and the DEM Sph(d18:0) was decreased (Figure 7C). Concurrently, in the BCAA degradation pathway, DEPs (IVD, ACAT2, HIBADH) were upregulated while L-3-Aminoisobutyric acid was decreased (Figure 7D).

## 4 Discussion

High-concentrate diets inherently challenge colonic integrity in beef cattle, driving microbial dysbiosis and systemic inflammation (Plaizier et al., 2021; Chen M. et al., 2023; Jin et al., 2024). Here, we show that AMC supplementation attenuated indices of colonic dysfunction. Our integrated multi-omics analysis revealed that these benefits were accompanied by coordinated shifts in the colonic microbiota, metabolome, and proteome, including changes in pathways linked to barrier function and electrolyte homeostasis. These findings suggest that AMC may be an effective nutritional strategy to support intestinal health in finishing cattle.

AMC supplementation altered the composition of the colonic microbiota, a community highly sensitive to dietary intervention (Zhang et al., 2021). Specifically, AMC supplementation enhanced microbial α-diversity, which has been associated with gut ecosystem stability and colonization resilience in various mammalian hosts (Sommer et al., 2017; Anzà et al., 2023). This contrasts with antibiotic growth promoters, which often reduce microbial richness and increase susceptibility to pathogen invasion (Thomas et al., 2017). In addition, AMC supplementation reduced the abundance of several taxa, including *Alphaproteobacteria* and *Mogibacterium*, some members of which have been reported to be associated with intestinal dysbiosis and inflammatory responses in previous studies (Spees et al., 2013; Marchesi et al., 2016; Horvath et al., 2021; Sun et al., 2021; Xu et al., 2024). The abundance of these taxa was positively correlated with the level of the pro-inflammatory cytokine IL-6. These findings are consistent with the concept that specific microbial signatures may reflect host inflammatory status (Schirmer et al., 2016). Furthermore, functional prediction suggested that these compositional changes were accompanied by a metabolic shift, notably the upregulation of microbial arginine biosynthesis, a pathway critical for immune regulation and inflammation control (Barrientos-Moreno et al., 2019).

Alongside the microbial changes, metabolomic analysis identified a significant reduction in Sph(d18:0) and ornithine in the AMC group. It is worth noting that sphingolipids are mainly synthesized by members of the *Bacteroidetes* and certain members of the *Alphaproteobacteria* (Zhao et al., 2023). In our study, the reduction in *Alphaproteobacteria* was concurrent with the decrease in Sph(d18:0), and a positive correlation was observed between them. Importantly, Sph(d18:0) was positively correlated with serum DAO and IL-6. While sphingolipids are typically recognized for supporting intestinal homeostasis (Brown et al., 2019), their accumulation under stress can be deleterious. This finding is consistent with the results of the mouse colitis model, that is, dysregulated sphingolipid metabolism can drive the host’s immune response (Doll and Snider, 2024). Specifically, sphingolipids like Sph(d18:0) are precursors of ceramides, which are recognized as negative regulators of intestinal health that can disrupt tight junctions, trigger pro-inflammatory cytokine production, and induce epithelial cell apoptosis (Li et al., 2018, 2022; Nagy-Szakal et al., 2018; Calzada et al., 2022; Thakkar et al., 2025). Therefore, the reduction in Sph(d18:0) is consistent with the suppression of this pro-inflammatory and pro-apoptotic pathway (Li et al., 2018; Thakkar et al., 2025). Beyond their role in mucosal inflammation, sphingolipids have been increasingly recognized as mediators of host-microbiome crosstalk in the gut. Commensal bacteria-derived sphingolipids can be sensed by host epithelial cells and modulate immune homeostasis (Heaver et al., 2018). The parallel changes in Alphaproteobacteria abundance and host sphingolipid profiles observed here are suggestive of such interkingdom signaling in the bovine colon, although direct causal demonstration requires further study. Additionally, the concurrent decrease in ornithine and predicted upregulation of arginine biosynthesis raises the possibility that metabolic flux may be directed toward polyamine synthesis, a pathway linked to enhanced intestinal barrier function (Deshpande et al., 2017; Zhang et al., 2025).

Joint analysis of the proteomic and metabolomic data identified coordinated changes in two pathways: sphingolipid signaling pathway and BCAA degradation. In the sphingolipid signaling pathway, key signaling proteins (PIK3CD, PIK3R1, BID, TRADD) were downregulated in the proteome, paralleling the reduction in Sph(d18:0) in the metabolome, suggesting a coordinated dampening of this pathway at both the protein and metabolite levels. In the BCAA degradation pathway, catalytic enzymes (IVD, ACAT2, HIBADH) were upregulated while L-3-aminoisobutyric acid, a pathway intermediate, was decreased, a pattern consistent with increased catabolic flux. Enhanced BCAA catabolism may support colonocyte energy metabolism and epithelial repair, while reducing substrates available for detrimental proteolytic fermentation (Nie et al., 2018; Gonsalves et al., 2020; Wu et al., 2022).

Beyond the pathway level changes described above, proteomic analysis also identified enrichment of pathways related to epithelial structure and function. The enrichment of the tight junction pathway was consistent with the upregulation of *ZO-1* and *MUC2*. Although Occludin, Claudin-1, and Claudin-4 were not altered at the mRNA level, this does not preclude changes in their protein expression or localization, which may occur through post-translational mechanisms not captured by qPCR (Shigetomi and Ikenouchi, 2018). The improvement in epithelial integrity and goblet cell density further supports a functional reinforcement of the mucosal barrier. The reduction in serum DAO, a marker of intestinal permeability, was consistent with improved barrier integrity (Liu et al., 2023). Crucially, a well-functioning intestinal barrier is a prerequisite for efficient electrolyte absorption, as the integrity of tight junctions and epithelial cell function are essential for the normal activity of ion transporters (Wu et al., 2019). In the colonic epithelium, sodium absorption is primarily driven by the basolateral Na^+^/K^+^-ATPase (encoded by *ATP1A1*), which establishes the electrochemical gradient necessary for apical sodium entry, while the electrogenic Na^+^-HCO_3_^+^ cotransporter *NBCe1* mediates basolateral sodium and bicarbonate influx (Yu et al., 2016; Negussie et al., 2022). The upregulation of *ATP1A1* and *NBCe1*, together with the water channel *AQP3*, is therefore consistent with enhanced sodium absorptive capacity and the osmotic gradient necessary for water reabsorption (Zhu et al., 2023). Furthermore, the enrichment of the aldosterone-regulated sodium reabsorption pathway suggests that AMC supplementation may promote colonic sodium transport, as aldosterone is a key regulator of ENaC and Na^+^/K^+^-ATPase expression in the intestinal epithelium (Rossier et al., 2015). The elevated serum K^+^ and Ca may reflect improved paracellular transport secondary to restored barrier integrity, although the specific mechanisms remain to be determined. Collectively, these results suggest that AMC supplementation may support colonic ion and water transport, potentially contributing to the observed improvement in fecal consistency.

Collectively, these findings indicate that AMC supplementation attenuated indices of colonic dysfunction, alongside coordinated shifts in the colonic microbiota, metabolome, and proteome. These beneficial effects were accompanied by changes in the composition of the colonic microbiota and alterations in both microbial and host metabolism, particularly involving the sphingolipid signaling pathway and BCAA degradation. These coordinated shifts were accompanied by reduced markers of inflammation and oxidative stress, alongside improvements in epithelial barrier integrity and absorptive capacity. It should be noted, however, that the present study cannot fully distinguish between effects directly attributable to the mineral and probiotic constituents of AMC and those resulting from the complex interplay among these components, the microbiota, and the host. The probiotic constituents contained in the AMC formulation may contribute to the microbial and metabolic shifts described above, while sodium and potassium may directly serve as substrates for ion transporters such as *ATP1A1* and *NBCe1*, and zinc may exert local anti-inflammatory effects (Gu et al., 2014; Goetz et al., 2023; Hu et al., 2023). Future studies using targeted inhibitors, germ-free models, or longitudinal omics designs will be needed to dissect the individual contributions of the probiotic and mineral components and their interactions with the colonic ecosystem.

This study has several limitations. Samples for omics and histological analyses were collected only at a single terminal time point (day 142); baseline and longitudinal measurements would have strengthened causal inference. The omics sample sizes were small, and histology was limited to 3 animals per group, which may reduce statistical power. Moreover, *P*-values for differential metabolites, proteins, and pathway enrichment were not adjusted for multiple comparisons; these results should therefore be viewed as exploratory. Because cattle were group-housed without individual feed bunks, the actual individual intake of AMC could not be verified, and the 20 g/d dose represents a target rather than a confirmed intake. The alkalinity and buffering capacity of AMC were not directly measured, nor were gastrointestinal or fecal pH evaluated. Finally, given the correlational nature of the multi-omics data, the observed associations among sphingolipid signaling, BCAA degradation, and host inflammatory and barrier indices remain to be causally validated in future studies.

## 5 Conclusion

**FIGURE 8:**
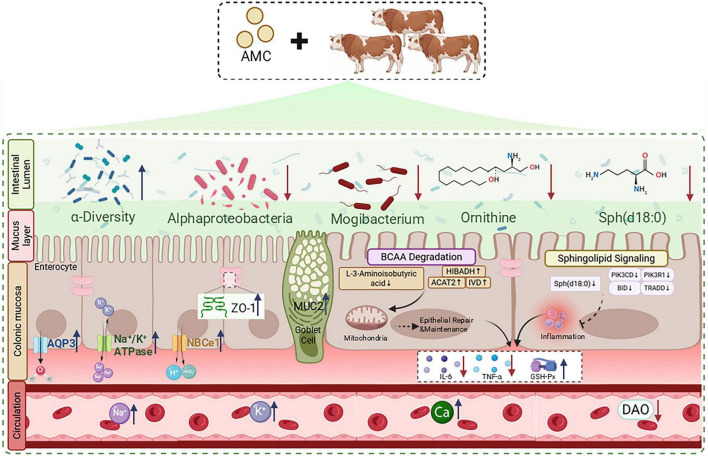
Improved colonic health induced by AMC supplementation is associated with changes in gut microbiota and host metabolism. AMC supplementation is associated with improved fecal consistency and markers of colonic health, accompanied by increased colonic microbial α-diversity, altered abundance of several bacterial taxa including *Alphaproteobacteria* and *Mogibacterium*, and lower levels of Sph(d18:0) and ornithine. These changes were observed alongside downregulation of key components of the sphingolipid signaling pathway (PIK3CD, PIK3R1, BID, TRADD) and upregulation of enzymes involved in BCAA degradation (IVD, ACAT2, HIBADH), as well as reduced markers of inflammation (TNF-α, IL-6), enhanced antioxidant defenses (GSH-Px), reinforcement of the epithelial barrier (*MUC2*, *ZO-1*, increased goblet cells), and improved ion and water transport (*AQP3*, *NBCe1*, *ATP1A1*). Dashed arrows denote associations inferred from integrated multi-omics analyses rather than proven causal relationships.

In summary, the results of this study suggest that AMC supplementation improves colonic health in finishing cattle, as evidenced by better fecal consistency, reduced inflammation, enhanced barrier integrity, and enhanced ion and water transport. These physiological improvements were accompanied by increased α-diversity and altered abundance of several bacterial taxa in the colonic microbiota, as well as by shifts in sphingolipid metabolism and BCAA degradation pathways. Integrated multi-omics analysis revealed coordinated changes in the gut microbiota, metabolome, and proteome that were correlated with the observed improvements in host physiology (Figure 8). Together, these findings suggest that AMC supplementation represents a promising nutritional strategy to support colonic function and mitigate diet-induced disturbances in finishing cattle. Future work should focus on validating the causal relationships underlying these associations and exploring the applicability of AMC supplementation across diverse production scenarios.

## Data Availability

The raw data of the 16S rRNA gene sequencing have been uploaded to the SRA database under the accession number PRJNA1346648 (https://dataview.ncbi.nlm.nih.gov/object/PRJNA 1346648?reviewer=86kd8nm57t58cc1h049gqb87vh). The raw data of the untargeted metabolomics has been uploaded to the MetaboLights database under the accession number MTBLS13173 (https://www.ebi.ac.uk/metabolights/MTBLS13173). The mass spectrometry proteomics data have been deposited to the ProteomeXchange Consortium via the iProX partner repository with the dataset identifier PXD069760 (https://proteomecentral.proteomexchange.org/?pxid=PXD069760).

## Footnotes

1
http://magic.novogene.com

2
https://biotree.lims2.com
