# Supplementary material for: Alkaline mineral complex supplementation alters gut microbiota and metabolic profiles and supports colonic health in finishing cattle
Source: Front Microbiol. 2026 Jun 2;17:1822268. doi: 10.3389/fmicb.2026.1822268 (PMC13269073; doi:10.3389/fmicb.2026.1822268)
Supplement: Supplementary file 1 [file Table_1.docx]

**Supplementary Data**

**Supplementary Table 1** The components and ion concentrations in AMC

| Microbial strain | Calculated Concentration (mg/kg) |
| --- | --- |
| *Bacillus subtilis* | 5 x 10^7^ CFU/g |
| *Bacillus licheniformis* | 1 x10^8^ CFU/g |

| Ions | Calculate Concentration (mg/kg) |
| --- | --- |
| Na^+^ | 27482.00 |
| K^+^ | 25103.00 |
| Zn^2+^ | 5.20 |
| Ge^4+^ | 0.13 |

**Supplementary Table 2** Composition and Nutrient Levels of Total Mixed Rations

| Diet Composition | Content (%) |
| --- | --- |
| DDGS (Distiller's dried grains with solubles) | 40.88 |
| Corn | 26.80 |
| Soybean meal | 1.81 |
| Premix ^①^ | 1.79 |
| Wheat bran | 1.83 |
| Rapeseed dregs | 1.86 |
| NaHCO_3_ | 0.80 |
| NaCl | 0.40 |
| Whole corn silage | 14.47 |
| Hay | 9.36 |
| Total | 100.00 |

| Nutrient levels | Content (%) |
| --- | --- |
| NEmf/(MJ/Kg) ^②^ | 1.44 |
| CP | 13.79 |
| NDF | 40.04 |
| peNDF | 19.65 |
| TDN | 64 |
| Ca | 0.29 |
| P | 0.33 |

Feed analyses were performed on the base TMR prior to AMC addition.

①Each kilogram of premix contains the following: 6,000 - 300,000 IU of Vitamin A, 6,000 - 90,000 IU of Vitamin D3, 200 mg of Vitamin E, 1,000 - 5,000 mg of Zinc, 1,000 - 6,000 mg of Iron, 275 - 750 mg of Copper, and 600 - 4,000 mg of Manganese. ②NEmf and TDN were calculated based on the Beef Cattle Feeding Standard (NY/T 815-2004), the others are measured values. ③Crude protein (CP) was determined using the Kjeldahl method. The NDF and peNDF contents were determined based on the methods described in the national standard for determination of neutral detergent fiber in feeds (GB/T20806-2022) and the local standard for determination of physically effective neutral detergent fiber in feeds (DB61/T 1063-2017), respectively. Calcium and phosphorus were analyzed using inductively coupled plasma optical emission spectrometry (ICP-OES) after wet ashing.

**Supplementary Table 3** Detailed Information of the Primers Used in the RT-qPCR Assay

| Gene Name | Primer Sequences (5’ to 3’) | Accession No. | Product Length (bp) |
| --- | --- | --- | --- |
| *Muc2* | F: TGCAGCATCATCAAAAGCCG  R: GTCGCAGAATATGGGGCACA | XM 024987595.1 | 216 |
| *Occludin* | F: GAACGAGAAGCGACTGTATC  R: CACTGCTGCTGTAATGAGG | NM_001082433.2 | 122 |
| *Claudin-1* | F: CGTGCCTTGATGGTGAT  R: CTGTGCCTCGTCGTCTT | NM_001001854.2 | 102 |
| *Claudin-4* | F: TCATCGGCAGCAACATCGTCAC  R: CAGCAGCGAGTCGTACACCTTG | XM_005892850.3 | 110 |
| *ZO-1* | R: TTGGACAAAGAGAAGGGTGAGA  F: AGACCAACCGTCAGGAGTCA | XM_024982007.2 | 129 |
| *AQP3* | F: AGCTGCCTGTGTACACCTTG  R: GGCCCAGATCGCATCGTAAT | NM_001079794.1 | 89 |
| *AQP4* | F: TACATTGCAGCTCAGTGCCT  R: ATTTCCGTGAACCGTGGTGA | NM_001317794.2 | 108 |
| *NHE3* | F: TCTCCGTGTACAGGGCCATA  R: GCCATAGCACATGACCACCT | XM_059878675.1 | 104 |
| *NBCe1* | F: AGGTGTGGACACTCCGAAAC  R: AACGGTGCAACAAACCAACC | NM_174605.1 | 81 |
| *ATP1A1* | F: AATGCGGAAGAGGTTGTCGT  R: TCTCCCGTGAGTGAGGAGTT | NM_001076798.1 | 122 |
| *β-actin* | F: CCATCGGCAATGAGCGGTTC  R: AGCACCGTGTTGGCGTAGAG | NM_173979.3 | 151 |

**Supplementary Table 4** Detailed results of quality control for 16S rRNA sequencing raw data.

| Sample | RawPE | Combined | Qualified | Nochime | Base(nt) | Avglen(nt) | GC(%) | Q20(%) | Q30(%) |
| --- | --- | --- | --- | --- | --- | --- | --- | --- | --- |
| Ctrl1 | 103320 | 97975 | 96630 | 85644 | 35295735 | 412.12 | 52.61 | 98.60 | 95.28 |
| Ctrl2 | 104017 | 103211 | 102206 | 76064 | 31445638 | 413.41 | 52.97 | 98.74 | 95.42 |
| Ctrl3 | 102318 | 101517 | 100423 | 85753 | 35070416 | 408.97 | 55.19 | 98.63 | 95.06 |
| Ctrl4 | 105654 | 105032 | 104009 | 98431 | 40208015 | 408.49 | 53.31 | 98.86 | 95.73 |
| Ctrl5 | 112781 | 111938 | 110828 | 86304 | 35414786 | 410.35 | 53.07 | 98.83 | 95.68 |
| AMC1 | 106411 | 105406 | 102703 | 72914 | 29684261 | 407.11 | 53.68 | 98.72 | 95.52 |
| AMC2 | 109572 | 108360 | 105378 | 67746 | 27921962 | 412.16 | 52.78 | 98.58 | 95.14 |
| AMC3 | 119328 | 117543 | 114425 | 91614 | 38088315 | 415.75 | 52.14 | 98.53 | 94.99 |
| AMC4 | 112889 | 112073 | 109451 | 88904 | 36788908 | 413.8 | 52.24 | 98.73 | 95.51 |
| AMC5 | 103889 | 103103 | 101959 | 73096 | 30222543 | 413.46 | 52.42 | 98.65 | 95.09 |

Raw PE: raw paired-end reads; Combined: tags obtained by merging paired-end reads; Qualified: high-quality tags after filtering low-quality bases and short sequences; Nochime: chimera-filtered tags (Effective Tags) used for downstream analysis; Base: total number of bases in Effective Tags; AvgLen: average length of Effective Tags; Q20 (%): percentage of bases with Phred quality score ≥ 20 (error rate < 1%); Q30 (%): percentage of bases with Phred quality score ≥ 30 (error rate < 0.1%); GC (%): GC content of Effective Tags.


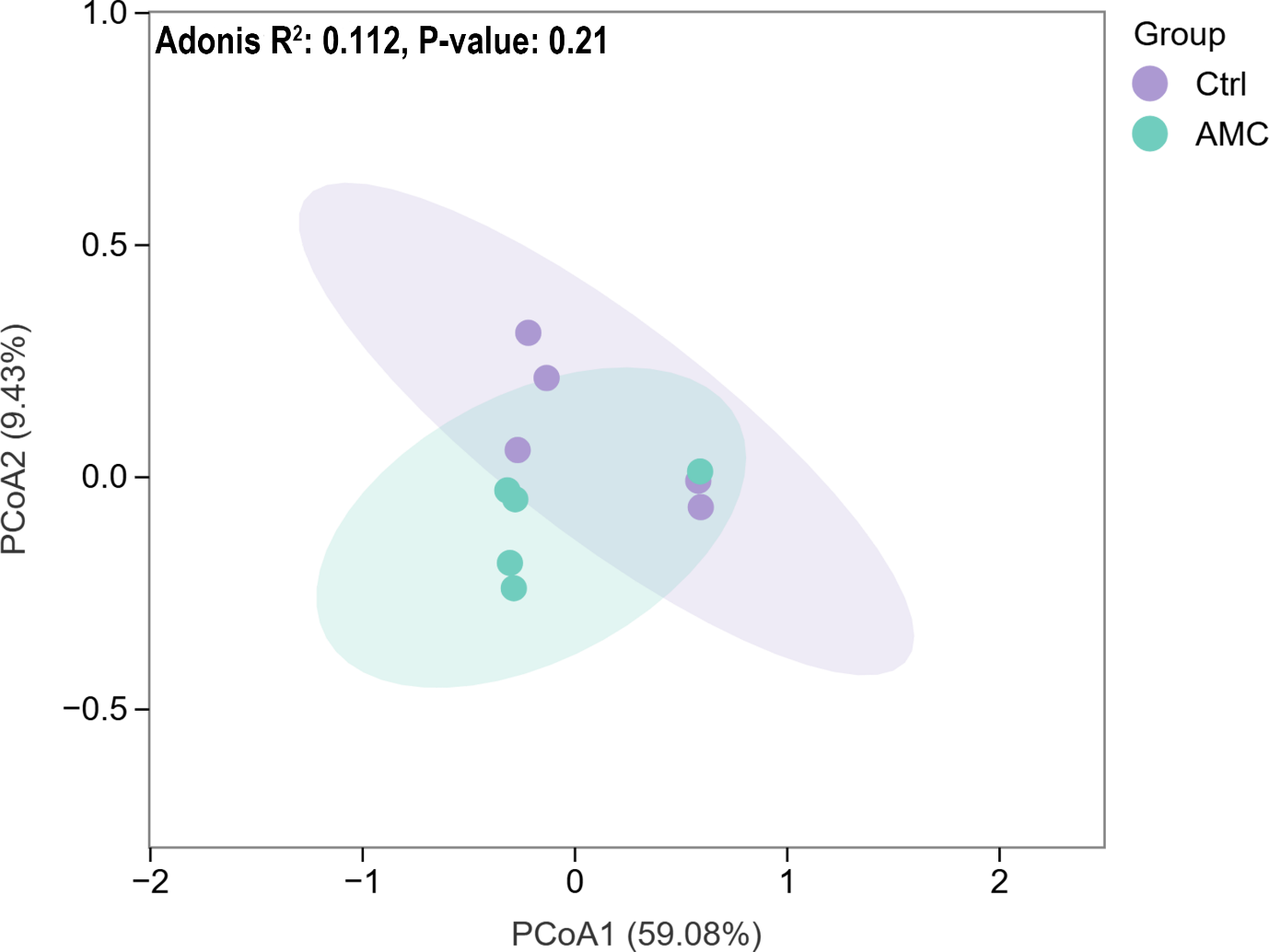


**Supplementary Figure 1** Principal coordinates analysis (PCoA)


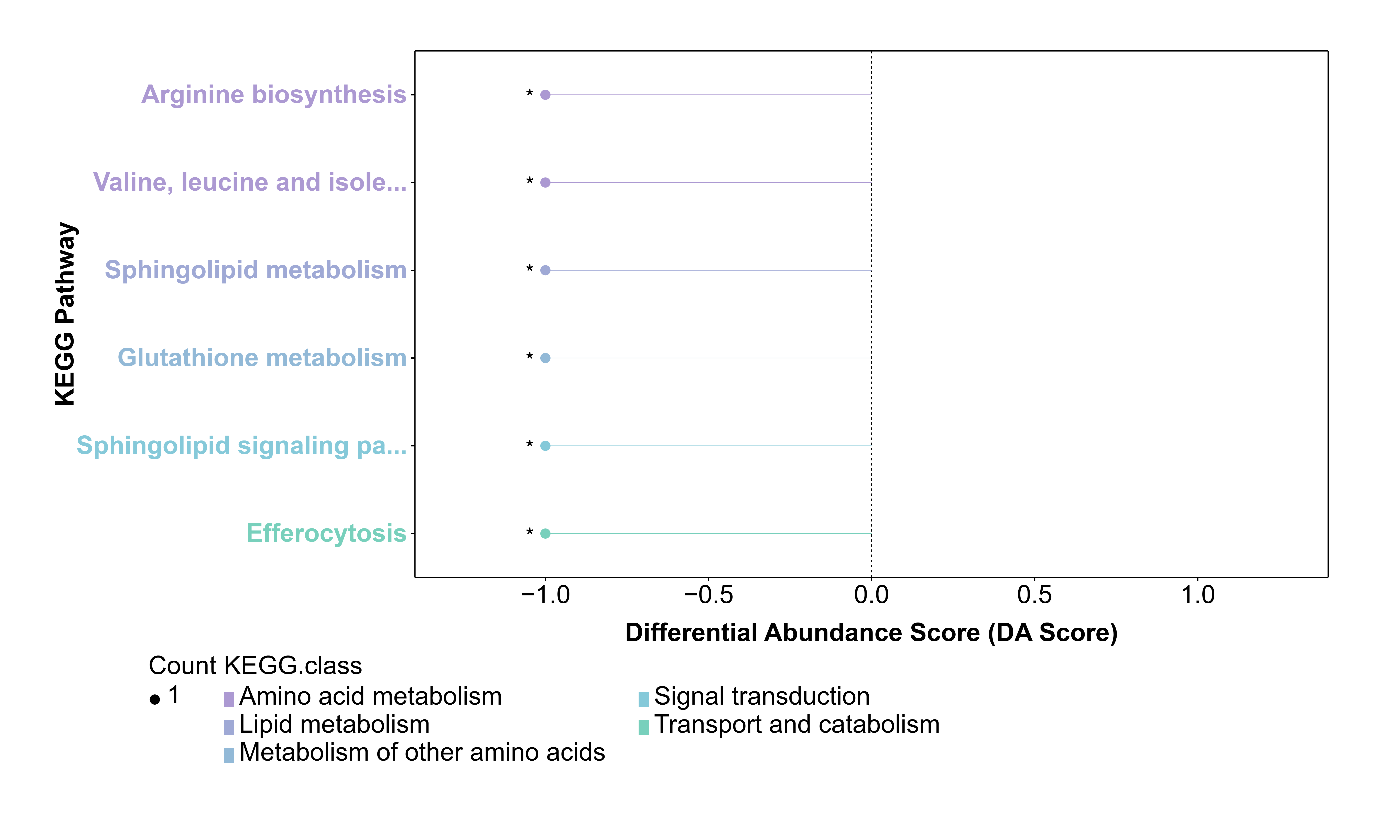


**Supplementary Figure 2** Differential abundance score (DAS)
